# eNd Points Challenges for Science and Policy

**DOI:** 10.1100/tsw.2001.458

**Published:** 2001-12-19

**Authors:** Johan Sliggers

**Affiliations:** Ministry of Housing, Spatial Planning and the Environment, The Hague, The Netherlands

**Keywords:** N-cycle, imbalance, reactive nitrogen, challenges, science, policy, integration, internalisation, globalisation, lifestyle, economic instruments, NitroGenius, Nitrogen Decision Support System (NDSS)

## Abstract

The theme of this Second International Nitrogen Conference is “Optimising nitrogen management in food and energy production and environmental protection”. With this theme in mind and with some observations, partly made during the week of the conference, some remarks can be made that will hopefully contribute to the goal of this conference and challenge and inspire both scientists and policy makers. Although being a representative of the Dutch government, the views and ideas brought forward in this paper do not necessarily represent those of the Dutch government. Also be aware of the fact that the ideas presented here are very much the views of a policy maker from a Western developed country.

The N-cycle is out of balance[1] and will continue to grow out of balance if we do not properly address the loss of reactive nitrogen. The demand for food and energy and thus the release of reactive nitrogen is enormous and will increase. The challenge we face is to bring this to a sustainable situation. The stakes are high and it is not a game. We are dealing with human health and the standard of living through the food and energy supply on the one hand and the quality of our environment with all the consequences for human health and nature on the other hand. In my view, the growing imbalance of the N-cycle is more serious and the clock is ticking more rapidly than for any other important environmental problem, including the problem of climate change. With this challenging statement a series of challenges for both scientists and policy makers will be laid out. The paper concludes with the objectives behind the N-game, NitroGenius.

## CHALLENGES FOR SCIENTISTS

As we have been discussing at this conference, surplus reactive nitrogen is not on the world’s political agenda. There are many problems with nitrogen compounds and many of them have been recognised as such for a long time. There are many efforts now being taken to address specific undesirable effects but there is no policy to close the N-cycle. The N-problems are not seen in the total context of closing this cycle. The first challenge therefore is to integrate our scientific understanding of nitrogen and to include other environmental issues such as climate change to show interactions (e.g., biodiversity, C and N fixation), to avoid shifts towards other problems or in time, and to demonstrate “win-win” situations (e.g., solar and wind energy, application of manure in favour of chemical fertilizer).

The second challenge is to give clear messages to policy makers: come down from science and explain the problems and possible solutions; use language the policy maker understands such as what are the costs to combat the problems and what are the benefits to be gained - if possible use monetary terms.

The third challenge is to base policy advice upon sound science: science on which there is consensus. Nothing is more confusing for policy makers and politicians—and more deadly for a cause—as quarrelling scientists. Of course there are more challenges one could think of but these are the most important ones in this context.

## CHALLENGES FOR POLICY MAKERS

For policy makers, the first challenge is to get the imbalance of the N-cycle on the political agenda. The approach to tackle the reactive nitrogen problem should be an integral policy. This integration does not only mean integration with the total nitrogen problem but also with other policy fields. Policy makers should work closely together with scientists to get decision makers interested and involved. Decision makers will only take action if this is the case. This is illustrated by the quadrant for scientists and policy makers (Fig. 1). Currently decision makers are in the lower left hand corner while they should be in the upper right hand corner. One way of getting them there is to make use of integrated assessment as has been done in Europe in the multieffects/multipollutants protocol, the UN/ECE Gothenburg protocol[2] under the Convention of Long-Range Transboundary Air Pollution.

Some have put forward the idea of a “Nitrogen Convention” just as for climate change. Preferably current institutions should integrate the loss of reactive nitrogen in their policies but a “Nitrogen Convention” could be a welcome addition to current institutions, provided it is integrated with these.

A problem that policy makers face is that policy is so slow. Not only does policy naturally lag behind science, it also seems almost impossible to get rid of old fashioned and out-of-date policy. For instance low cost food and low cost energy policies in the Western world are still very dominant, even though these policies literally stink. Such policies leads to intensified production with quantity emphasized over quality, low profits per unit of product, overuse of energy, water, nitrogen, and thus the deterioration of our planet. In this respect the Western world has strange lifestyles or preferences. Is eating more and more meat the way we do really healthy? Do we really prefer cheap energy to breathing fresh air? Is it normal that in many countries gasoline is cheaper than bottled drinking water? And should the whole world follow this example? The second challenge for policy makers would therefore be to internalise environmental costs into the price of products because at the moment the environment pays the price for cheap energy and cheap food. Of course this will reduce our present standard of living to a certain extent but not much and the Western world can afford it. Currently the environment is not incorporated in our economic figures such as the GDP. The result is that environment and economy often oppose each other instead of reinforcing one another.

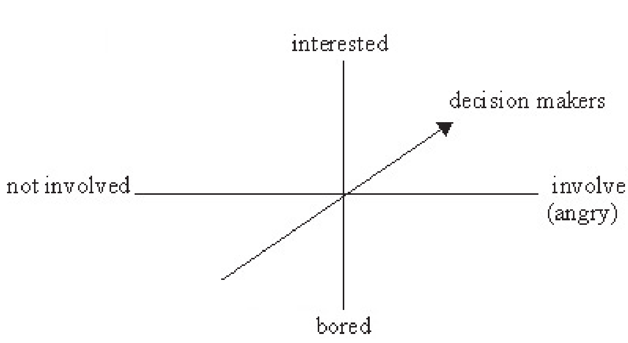

**FIGURE 1**. Quadrant for scientists and policy makers.

The third challenge is related to the second one: to minimize the negative effects of the global economy. The global economy determines many things. The difference of costs of labour around the world, the cheap transportation costs of goods and not taking into account the deterioration of our environment in the price of products lead to further inequities and more pollution. In the context of nitrogen an obvious example is intensive livestock farming far away from the production of fodder but there are many more examples. In the Netherlands we catch shrimp in the North Sea, put them into a truck, drive them to Maroc, where labour is cheap, to have them peeled and then transport them back to the Netherlands to be sold as fresh(?) shrimp. If we want to put a halt to this kind of behaviour WTO-GATT or EU rules often prevent us. Perhaps it is for these reasons an increasing number of people question the blessings of globalisation. Some people are bored and frustrated as can be seen at meetings of WTO, EU, G-8 etc. Clearly this group is in the lower right corner of the quadrant.

Over to the fourth and last challenge for policy makers, which is the implementation of policies and instruments. In the Western world money really is not the issue. We can afford to take measures. What we cannot afford is to go on conducting business as usual. The question is rather: are we prepared to invest in improving the quality of food, environment, and health? Since we are living in an economically driven society, we might as well make use of that. The internalisation of environmental costs has already been mentioned. Together with instruments such as restrictions, command and control, education, etc., economic incentives can be very powerful. Economic instrument such as levies, charges, subsidies, cap and trade systems, will become more and more important. At this conference ideas have come up such as charges on nitrogen in fertiliser and fodder, paying the price for sustainable produced animal feed and so on. But the possibilities go far beyond this: create a price differentiation in which less polluting products are favoured over more polluting products; shift taxation from labour to energy use, nitrogen use or emissions to the environment; make transport and energy more expensive; create conditions for better and realistic prices for sustainably produced products.

## NitroGenius (the N-Game)

The Dutch government has put quite some resources into a game, NitroGenius[1]. What are the reasons for developing this N-game? The ultimate goal is to get decision makers in the top right hand corner of the quadrant. The N-game is an instrument that can help to do just that. People who play the game experience the complexity of reactive nitrogen and learn to think integratively to find optimal solutions. Another thing the game does is let players assume other roles so they get a better understanding of the position of other stakeholders. Players are rewarded for finding “win-win” solutions.

We will not only use the game here at the Second Nitrogen Conference but we will also invite all major key stakeholders in the Netherlands to play the game to move them towards the top right hand corner of the quadrant. To build upon that we are developing a tool for stakeholders with which they can put together policy scenarios to enable them to see the results for environment and society. This is not a game anymore. It comes close to decision-making and taking action. That is why we have called it the Nitrogen Decision Support System (NDSS)[1]. We plan to put both the N-game and the NDSS on our website[3] so all participants of the Second Nitrogen Conference and other interested people can use them.

| | | |
|---|---|---|
| Received: | November | 18, 2001 |
| Revised: | November | 19, 2001 |
| Accepted: | November | 19, 2001 |
| Published: | December | 19, 2001 |
